# The Three Lipocalins of Egg-White: Only Ex-FABP Inhibits Siderophore-Dependent Iron Sequestration by *Salmonella* Enteritidis

**DOI:** 10.3389/fmicb.2020.00913

**Published:** 2020-05-15

**Authors:** Louis Alex Julien, Clémence Fau, Florence Baron, Sylvie Bonnassie, Catherine Guérin-Dubiard, Françoise Nau, Michel Gautier, Kimon Andreas Karatzas, Sophie Jan, Simon Colin Andrews

**Affiliations:** ^1^School of Biological Sciences, University of Reading, Reading, United Kingdom; ^2^STLO, INRAE, Institut Agro, Rennes, France; ^3^Inserm 1107, Neuro-Dol, Université Clermont Auvergne, Clermont-Ferrand, France; ^4^UFR Sciences de la Vie et de l’Environnement, Université de Rennes I, Rennes, France; ^5^Food and Nutritional Sciences, University of Reading, Reading, United Kingdom

**Keywords:** salmochelin, enterobactin, Ex-FABP, Cal-γ, α-1-ovoglycoprotein, egg white, *Salmonella* Enteritidis

## Abstract

*Salmonella* Enteritidis is the most prevalent food-borne pathogen associated with egg-related outbreaks in the European Union. During egg colonization, *S.* Enteritidis must resist the powerful anti-bacterial activities of egg white (EW) and overcome ovotransferrin-imposed iron-restriction (the most important anti-bacterial mechanism of EW). Many pathogens respond to iron restriction by secreting iron-chelating chemicals called siderophores but EW contains a siderophore-sequestering “lipocalin” protein (Ex-FABP) that is predicted to limit the usefulness of siderophores in EW. *S.* Enteritidis produces two siderophores: enterobactin, which is strongly bound by Ex-FABP; and the di-glucosylated enterobactin-derivative, salmochelin (a so-called “stealth” siderophore), which is not recognized by Ex-FABP. Thus, production of salmochelin may allow *S.* Enteritidis to escape Ex-FABP-mediated growth inhibition under iron restriction although it is unclear whether its EW concentration is sufficient to inhibit pathogens. Further, two other lipocalins (Cal-γ and α-1-ovoglycoprotein) are found in EW but their siderophore sequestration potential remains unexplored. In addition, the effect of EW lipocalins on the major EW pathogen, *S.* Enteritidis, has yet to be reported. We overexpressed and purified the three lipocalins of EW and investigated their ability to interact with the siderophores of *S*. Enteritidis, as well as their EW concentrations. The results show that Ex-FABP is present in EW at concentrations (5.1 μM) sufficient to inhibit growth of a salmochelin-deficient *S.* Enteritidis mutant under iron restriction but has little impact on the salmochelin-producing wildtype. Neither Cal-γ nor α-1-ovoglycoprotein bind salmochelin or enterobactin, nor do they inhibit iron-restricted growth of *S.* Enteritidis. However, both are present in EW at significant concentrations (5.6 and 233 μM, respectively) indicating that α-1-ovoglycoprotein is the 4th most abundant protein in EW, with Cal-γ and Ex-FABP at 11th and 12th most abundant. Further, we confirm the preference (16-fold) of Ex-FABP for the ferrated form (K_*d*_ of 5.3 nM) of enterobactin over the iron-free form (K_*d*_ of 86.2 nM), and its lack of affinity for salmochelin. In conclusion, our findings show that salmochelin production by *S.* Enteritidis enables this key egg-associated pathogen to overcome the enterobactin-sequestration activity of Ex-FABP when this lipocalin is provided at levels found in EW.

## Introduction

From 2010 to 2013, the number of confirmed human salmonellosis cases in the European Union (EU) decreased by ∼15% (Efsa Panel on Biological Hazards, 2019) but increased again after 2014 (Efsa Panel on Biological Hazards, 2019; ECDC and EFSA, 2017) with more than 750 strongly-evidenced food-borne *Salmonella* outbreaks reported between 2014 and 2016. Of these cases, eggs and egg products were identified as the main vehicle of infection (276 cases). In comparison, broiler meat (*Gallus gallus*) and derived meat products were responsible for only 46 such outbreaks (Efsa Panel on Biological Hazards, 2019). Together, *Salmonella enterica* serovars Enteritidis (*S*E) and Typhimurium were the most frequently detected in food-borne outbreaks linked with egg and egg products in the EU (92 and 5.9% of outbreaks, respectively), with *S*E being by far the most prevalent serovar (Efsa Panel on Biological Hazards, 2019). This indicates that, despite the current controls, *S*E infection of eggs remains a serious threat to health in the EU (Efsa Panel on Biological Hazards, 2014; ECDC and EFSA, 2017; Efsa Panel on Biological Hazards, 2019).

The two possible routes of egg contamination are horizontal and vertical transmission. The former refers to penetration through the eggshell while the latter results from the infection of reproductive organs (Gantois et al., 2009). During egg formation, *S*E contamination of both the yolk and the egg white (EW) have been reported (Gantois et al., 2009 for a review). Although there is no clear consensus in the literature, it has been suggested that the albumen is the more likely site of initial infection than the yolk (Gast and Beard, 1990; Humphrey et al., 1991; Keller et al., 1995). During horizontal transmission, colonization and survival in EW also seem crucial as *S*E penetrates through the shell and encounters this medium prior to the vitelline membrane and the yolk.

Egg white carries a powerful set of antimicrobial activities that *S*E must resist in order to mediate egg-linked infections (Clavijo et al., 2006; Vylder et al., 2013; Baron et al., 2016). The antibacterial properties of EW include its high pH and viscosity, and a diverse range of antibacterial proteins/peptide (Baron et al., 2016). However, the major factor limiting bacterial growth in EW is iron restriction (Schade and Caroline, 1944; Garibaldi, 1970; Lock and Board, 1992; Baron et al., 1997). Iron restriction arises from the high levels (∼170 μM; second most abundant EW protein) of ovotransferrin (oTf; also known as conalbumin; Alderton et al., 1946), a powerful Fe^3+^-chelating protein (binding constant of 10^32^ M^−1^; Chart and Rowe, 1993). OTf is closely related to serum Tf which delivers iron between tissues in man and other vertebrates (Williams, 1968). The iron-binding capacity of oTf outstrips the iron content of EW (0.1 mg iron per 100 g; Nys and Sauveur, 2004; USDA, 2010) by ∼20-fold which suggests that there is virtually no “free” iron in EW (Julien et al., 2019). This high oTF:Fe ratio of EW resembles the conditions of serum where the partial iron saturation of circulating Tf (∼30%; Kelly et al., 2017) causes a powerful bacteriostatic iron-restriction effect (Bullen et al., 1978).

An important mechanism used by bacteria to overcome iron restriction involves the secretion of high-affinity ferric-iron-chelating molecules called siderophores (Andrews et al., 2003). *S*E produces two related siderophores, enterobactin (Ent) and salmochelin (Sal), both of which are catecholates (Pollack et al., 1970). Ent is a serine macrotrilactone and Sal is its glucosylated derivative. Hence the term Sal can refer to both mono-glucosylated Ent (MGE) and di-glucosylated Ent (DGE). Ent is reported as the strongest siderophore known, binding to ferric ion with an affinity constant of 10^52^ M^−1^ (Chart and Rowe, 1993). Because Ent has a higher affinity for iron than oTf/Tf, Ent-producing bacteria can acquire iron from both oTf and Tf (Ford et al., 1988; Carrano and Raymond, 1979; Chart and Rowe, 1993). The genetic locus responsible for this glucosylation of Ent in *Salmonella* is the *iroBCDEN* gene cluster (Bäumler et al., 1998). The di-glucosylation of Ent to generate Sal is catalyzed by the glucosyltransferase, IroB (Hantke et al., 2003; Bister et al., 2004). The resulting Sal is then exported across the cytosolic membrane by IroC (Crouch et al., 2008). Uptake and utilization are mediated by IroD, IroE, and IroN (Müller et al., 2009 for a review). Although the affinity of Sal for Fe^3+^ is not reported (Valdebenito et al., 2007; Watts et al., 2012), there is no indication that glucosylation significantly impacts Fe^3+^ ligation or affinity (Luo et al., 2006).

The host immune system can counter the action of bacterial siderophores through release of siderophore-binding proteins that remove ferri-siderophores from circulation (Clifton et al., 2009). These proteins belong to the lipocalin superfamily which includes members with a diverse range of functions beyond siderophore sequestration (Johnstone and Nolan, 2015). Human lipocalin 2 (LCN2) is the best-known example of a siderophore-binding lipocalin; it is able to sequester a variety of bacterial siderophores, including Ent (Goetz et al., 2002; Holmes et al., 2005). However, many bacteria can overcome the siderophore-sequestration activities of LCN2 by producing “stealth” siderophores, such Sal, which are not recognized by LCN2 (Fischbach et al., 2006; Valdebenito et al., 2007).

In EW, three lipocalin proteins have been identified: the extra fatty acid binding protein (Ex-FABP, or Ch21); the chondrogenesis-associated lipocalin (Cal-γ or prostaglandin D synthase); and α-1-ovoglycoprotein (Guérin-Dubiard et al., 2006; Mann, 2007; D’Ambrosio et al., 2008; Mann and Mann, 2011). Of these three EW proteins, siderophore-binding function has only been explored for Ex-FABP. Ex-FABP was found to have similar function to LCN2 in sequestering ferric-Ent with high affinity (equilibrium dissociation constant, K_*d*_, of 0.22 nM; Correnti et al., 2011). Ex-FABP also inhibits iron-restricted growth of a strain of *Escherichia coli* producing Ent as sole siderophore (Correnti et al., 2011), but not strains producing Sal (Garénaux et al., 2013). However, it is unclear whether Ex-FABP is present in EW at concentrations sufficient to inhibit growth of Ent-producing bacteria. Moreover, it is unclear whether the two other lipocalins of EW (Cal-γ and α-1-glycoprotein) might also sequester siderophores. Further, the capacity of Ex-FABP to inhibit the key EW-mediated pathogen, *S*E, under iron restriction has yet to be explored.

The aim of the research reported here was to explore whether the three lipocalins of EW can sequester the two siderophores produced by *S*E and to determine whether the EW lipocalins can inhibit *S*E growth under the iron-restricted conditions. The results show that only Ex-FABP inhibits iron restricted growth of *S*E and binds Ent with high affinity, and that it is present in EW at levels sufficient to inhibit growth when Ent is the sole siderophore produced. The results reported thus suggest that Ex-FABP is an effective component of the antibacterial repertoire of EW.

## Materials and Methods

### Growth Media, Strains, and Plasmids

Lysogeny broth (LB) was used as the growth medium for *E. coli*, whereas either LB or Tryptone Soy Broth (TSB, Sigma-Aldrich) were used for the growth of *S. enterica*. M9 salts (Sigma-Aldrich) minimal medium (with 1 mM MgSO_4_.7H_2_O, 0.2% w/v glucose, 20 μM thiamine, 0.1 mM CaCl_2_, 0.3% w/v casamino acids) and acid washed glassware were used to achieve iron-restricted growth. Ferric citrate (10 μM) was added to achieve iron sufficiency. Growth in liquid medium was at 37°C with shaking generally at 250 rpm. *E. coli* TOP10 was used for cloning work and *E. coli* K-12 BW25113 Δ*entB*::*kan* (λDE3) was used as overexpression host. *S. enterica* serovar Enteritidis PT4-P125109 was used for growth tests and gene knockout. The bacterial strains and plasmids used and generated are listed in Tables 1, 2. Siderophore detection assay was performed using CAS (chrome azurol S) plates according to Louden et al. (2011).

**TABLE 1:** Bacterial strains used and their genetic features.

Strain name	Features	Source
*E. coli* Top10	F^–^ *mcrA* Δ(*mrr-hsdRMS-mcrBC*) Φ80*la*cZΔM15 Δ*lacX74 recA1 araD139* Δ(*ara leu*)7697 *galU galK rpsL* (Str^*R*^) *endA1 nupG*	Invitrogen
BW25113	*E. coli* K-12	GE Healthcare
JW0587(λDE3)	BW25113 (Δ*entB*)::*kan* (λDE3)	This study
PT4-P125109	Wild type *Salmonella enterica* serovar Enteritidis PT4-P125109	McCusker Dublin, Ireland
PT4-P125109 Δ*entB*	*Salmonella enterica* serovar Enteritidis PT4-P125109 Δ*entB*	This study
PT4-P125109 Δ*iroBC*	*Salmonella enterica* serovar Enteritidis PT4-P125109 Δ*iroBC*	This study
PT4-P125109 Δ*iroDEN*	*Salmonella enterica* serovar Enteritidis PT4-P125109 Δ*iroDEN*	This study

**TABLE 2:** Plasmid used and their features.

Plasmid	Size (bp)	Features	Resistance	Source
pMK-lcn2	2930	Encodes LCN2	Kan	GeneArt synthesis (Invitrogen)
pMK-Ex-FABP	2860	Encodes Ex-FABP	Kan	GeneArt synthesis (Invitrogen)
pMK-Cal-γ	2881	Encodes Cal-γ	Kan	GeneArt synthesis (Invitrogen)
pMK-α1-glyco	2935	Encodes α1-glycoprotein	Kan	GeneArt synthesis (Invitrogen)
pET21a	5443	Requires T7 RNA polymerase and under LacUV5 control	Amp	Novagen
pET-lcn2	5904	pET21a vector used for LCN2 over-production	Amp	This study
pET-Ex-FABP	5907	pET21a vector used for Ex-FABP over-production	Amp	This study
pET-Cal-γ	5928	pET21a vector used for CAL-γ over-production	Amp	This study
pET-α1-glyco	5982	pET21a vector used for α-1-glycoprotein over-production	Amp	This study
pKD3	2804	λ red template vector	Cm, Amp	Datsenko and Wanner, 2000
pKD46	6329	T°C sensitive (30°C) λ red expressing vector encoding *exo, beta* and *gam* and under AraC control	Amp	Datsenko and Wanner, 2000
pCP20	9332	T°C sensitive (30°C) vector encoding FLP recombinase	Cm, Amp	H. Mori, Japan

### Inactivation of Genes Required for Enterobactin and Salmochelin Production/Utilization

The *entB* (enterobactin biosynthesis), *iroBC* (salmochelin uptake and synthesis), and *iroDEN* (salmochelin uptake and utilization) genes of *S*E were inactivated using the λRed gene disruption system encoded by plasmid pKD46 (Datsenko and Wanner, 2000; Supplementary Figure S1 and Supplementary Table S1). The chloramphenicol resistance cassette of pKD3 inserted in the genome was subsequently deleted using FLP recombinase encoded by the temperature-sensitive plasmid, pCP20 (Cherepanov and Wackernagel, 1995) to generate clean deletions. Mutants were confirmed by whole-genome sequencing (Illumina; MicrobesNG, Birmingham); corresponding genome sequences were deposited at the ENA database under the following project accession number: PRJEB36543. General molecular genetic methods were as previously described (Sambrook and Russell, 2001).

### Cloning of Lipocalin Genes for Over-Production in *E. coli*

Nucleotide sequences for the human LCN2 and chicken Ex-FABP, α-1-ovoglycoprotein and Cal-γ genes were obtained from the NCBI GeneBank database using the ID numbers 3934, 396393, 395220, and 374110, respectively. The sequences were codon optimized for expression in *E. coli* (GeneArt optimisation portal;^1^) (see Supplementary Figure S2 for corresponding sequences) and then synthesized by GeneArt (Invitrogen, Germany) with flanking restriction sites to assist subsequent cloning from the pMK carrier plasmid together with an N-terminal signal sequence (except for LCN2, which does not have an N-terminal signal sequence) to allow secretion of the encoded hexa-His tagged proteins into the periplasm. The lipocalin-coding regions were released from the pMK plasmid using *Nde*I and *Xho*I, and then ligated into the corresponding sites of plasmid pET21a using the Rapid DNA Ligation kit (Thermo Fisher Scientific). The resulting constructs were confirmed by restriction digestion and nucleotide sequencing (Eurofins). *E. coli* K-12 BW25113 Δ*entB*::*kan* (JW0587) was converted to λDE3-lysogen status using a Lysogenization kit (Novagen) and the resulting strain then acted as host for over-expression of the lipocalin genes from pET21a. This enterobactin-deficient host strain was used to avoid association of overexpressed lipocalins with host-specified enterobactin.

### Lipocalin Purification

**FIGURE 1:**
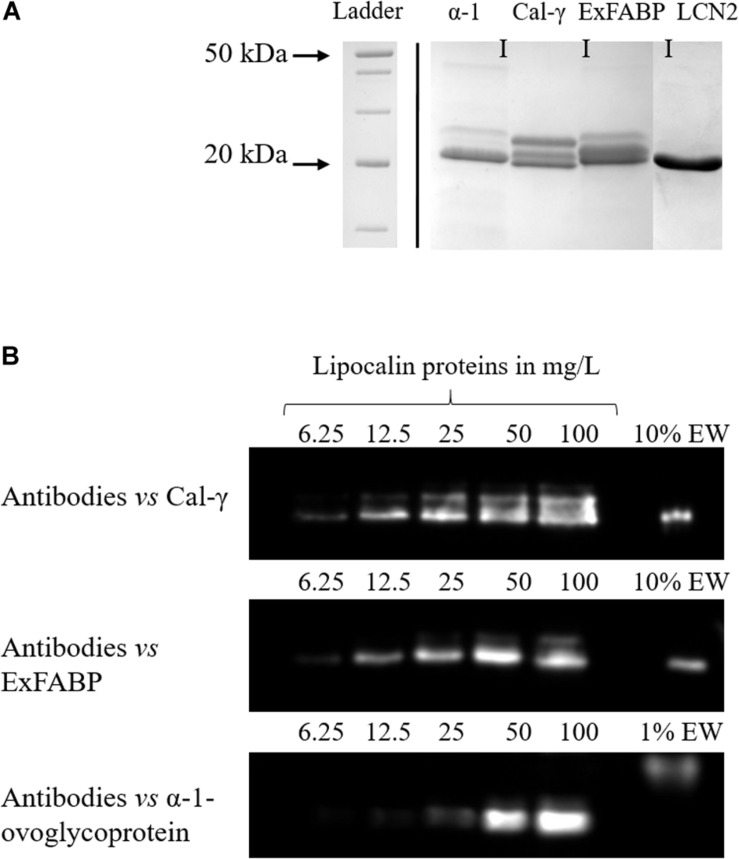
Purification of lipocalins **(A)** and quantification of lipocalin levels in EW **(B)**. **(A)** SDS-PAGE analysis of overproduced, purified EW lipocalins. **(B)** Western-blot analysis of a range of EW lipocalin levels (as indicated) as well as a sample of EW (diluted 1:10 or 1:100 in dH_2_O). The calibration curves of purified EW lipocalins used to calculate the amounts found naturally in EW are provided in the Supplementary Figure S3.

*Escherichia coli* JW0587(λDE3) transformants, carrying a corresponding lipocalin-expressing pET21a plasmid, were grown in LB (supplemented with 10 μM Fe-citrate) and expression was induced with 0.5 mM IPTG. Cells pellet were harvested by centrifugation (3000 × *g* for 20 min at 4°C), resuspended in 1 mL of osmotic shock solution (20% sucrose, 30 mM Tris pH 8.0, 10 mM EDTA) per 50 mg of cells (∼5 g in total). After 10 min at room temperature, the supernatant was harvested as above and dialysed thoroughly against binding buffer 1 (0.3 M NaCl, 50 mM Na_3_HPO_4_, 15 mM imidazole, pH 8.0) at 4°C. The C-terminally hexa-His-tagged lipocalin proteins were isolated from the retentate by Ni^2+^-affinity chromatography using a 5 mL Mini Nuvia IMAC Ni-charged cartridge (Bio-Rad) according to manufacturer’s instructions. Eluted Ex-FABP and α-1-ovoglycoprotein were further purified using a 5 mL HiScreen Capto Diethylaminoethyl column (DEAE-sepharose, GE Healthcare) equilibrated in binding buffer 2 (20 mM Tris–HCl, pH 8) with elution achieved with a linear gradient of 1 M NaCl in the same buffer. LCN2 and Cal-γ were further purified using a 5 mL HiScreen Capto Multimodel Cation exchanger ImpRes column (MMC, GE Healthcare) equilibrated with binding buffer 3 (50 mM sodium acetate, pH 6); elution was achieved with a linear gradient of 1 M NaCl in the same buffer or in Tris–HCl at pH 8. The purity of the resulting proteins was determined by SDS-PAGE (Sambrook and Russell, 2001; Figure 1A) and identities were confirmed by electrospray-ionisation mass-spectrometry (ESI-MS). Bradford protein assay (Bio-Rad) and absorbance at 280 nm were used to estimate protein concentrations. Proteins were stored in phosphate-buffered saline (PBS, pH 7.4) at −80°C.

### Electrospray Ionization – Mass Spectrometry

Isolated lipocalin proteins were dialysed against ultrapure water prior to analysis by MicroTOF-Q mass spectrometry (column Ace C8 50 × 2.1, mass spectrometer Thermo Scientific LTQ Orbitrap XL with an ESI source *via* a Thermo Scientific Accela HPLC). Mass spectrum peaks were averaged and deconvolution to uncharged neutral mass was performed using Xtract within the Xcalibur software (the BioCentre, University of Reading).

### Western-Blot Analysis of Lipocalin Levels in EW

Polyclonal antibodies were raised in two New Zealand White rabbits by DC Biosciences (Dundee, United Kingdom) using 2–2.5 mg of each of the three purified chicken-EW lipocalin proteins. A 90-day protocol using Freud’s adjuvant as a stimulating agent was followed. The levels of each lipocalin in EW were then determined by western blotting (Mahmood and Yang, 2012) using corresponding lipocalin standards (6.25; 12.5; 50; 100 mg/L) along with EW dilutions (1:10 or 1:100 in qH_2_O). Immunodetection was achieved using the primary antibodies raised in rabbit, and secondary anti-rabbit IgG antibody raised in goat and HRP-conjugated (Sigma-Aldrich batch A-6154). Westar C 2.0 chemiluminescent substrate (Cyanagen) was used for signal development. The membrane was imaged using a Syngene G:BOX. Images were analyzed with ImageJ software in order to band determine signal intensities. The lipocalin quantification was repeated three times with eggs of three different egg brands (free range eggs, the Cooperative; free range eggs, Clarence Court; and free-range eggs, Happy Egg). All eggs were certified Class A and produced in the United Kingdom. From the three concentration values gathered, an average and standard error were calculated.

### Isothermal Titration Calorimetry

Experiments were carried out with a GE Healthcare Microcal VP-ITC microcalorimeter according to the manufacturer’s guidelines (Operating Instructions 28-9639-79 AA). The device consisted of a motorized injection syringe, in which the ligand was loaded, with two adiabatic cells set at 30 °C. The left cell was filled with the reference (TBS with 1.3% DMSO), whereas the right cell was filled with 5 μM lipocalin protein dissolved in the same buffer. Sequential 10 μL injections (29 in total) of ligand were applied consisting of either 50 μM enterobactin (Sigma-Aldrich) or 50 μM salmochelin (EMC siderophore) dissolved in TBS (pH 7.4) with 1.3% DMSO. Samples were degassed and pre-equilibrated at the desired temperature using a GE MicroCal ThermoVac degassing station before loading into the syringe, sample cell and reference cell.

### Growth Tests in Liquid Medium

Growth comparison in iron-free M9 medium (with/without 10 μM ferric citrate) was performed in 100-deepwell Honeycomb plates incubated in a Bioscreen C (Lab System) apparatus (at 37°C with constant shaking at 200 rpm) for ∼20 h. Growth comparison in LB medium (with/without 200 μM 2,2 dipyridyl; DIP; an iron chelator) was achieved using 96-well microplates incubated in a Spectrophotometer SpectraMax 340 PC (Molecular devices) at 37°C with 60 s shaking at 200 rpm every 15 min (for ∼20 h).

## Results and Discussion

### Isolation of EW Lipocalins

The genes encoding the three EW lipocalins (Ex-FABP, Cal-γ, and α1-glycoprotein) and human lipocalin 2 (LCN2) were codon-optimized (for *E. coli*) and overexpressed in *E. coli* to give C-terminal hexa-His-tagged recombinant proteins, as described in Experimental Procedures. Purification was then achieved using Ni^2+^-affinity and ion-exchange chromatography. SDS-PAGE analysis indicated a purity of ≥90% and polypeptides of the anticipated mobility (Figure 1A). ESI-MS of α-1-ovoglycoprotein and Ex-FABP showed that, in each case, the major polypeptide was a close match to the theoretical mass of the mature forms (Table 3), indicating cleavage of the PelB_*ss*_ had been achieved during secretion. However, lower levels of the immature form of α-1-ovoglycoprotein were recovered (Figure 1A and Table 1). For Cal-γ, ESI-MS indicated a mass that is 763 Da greater than that predicted for both the major mature and minor immature forms. ESI-MS analysis carried out under native and denaturing conditions showed that the additional mass was likely covalently association, and MALDI-ISD showed that the N- and C-terminal amino acid sequences (residues 1–36 and 161–173) of the mature form were as expected. Thus, the nature of the additional 763 Da component remains unclear.

**TABLE 3:** Comparison of the purification products detected through ESI-MS and the theoretical mass of the lipocalin-like proteins.

	Mass determined	Theoretical	Difference between
	through	mass	the theory and the
	ESI (Da)	(Da)	observed Mass (Da)
Mature α1-glyco	21,377.5	21,381.9	4.4
PelB-α1-glyco	23,586.7	23,592.7	5.9
Mature Ex-FABP	19,127.4	19,130.6	3.2
Mature Cal-γ	20,655.0	19,892.2	762.8
PelB-Cal-γ	22,866.2	22,103.0	763.2

### EW Contains Micro-Molar Levels of All Three Lipocalins

The purified EW lipocalins were used to raise antibodies in rabbit to enable quantification of the lipocalins in EW by western blotting (Figure 1B). Western-blot analysis showed that all three lipocalins are indeed present in EW at detectable levels, with mobilities matching those of the purified proteins for Ex-FABP and Cal-γ (Figure 1B). However, α-1-ovoglycoprotein in EW showed a higher apparent mass (37.4 kDa) than that of the recombinant protein (22.7 kDa). Haginaka et al. (1995) also reported a higher mass for α-1-ovoglycoprotein in EW by MALDI-TOF mass spectroscopy (30 kDa). These discrepancies are presumed to arise from the N-type glycosylation of α-1-ovoglycoprotein within EW (30.3% total glycosylation; Ketterer, 1965) resulting in aberrant mobility of glycosylated proteins during SDS-PAGE (Guérin-Dubiard et al., 2006). The semi-quantitative western blots indicated EW concentrations of 232.9, 5.6, and 5.1 μM for α-1-ovoglycoprotein, Cal-γ, and Ex-FABP, respectively (Table 4). They are therefore the 4th, 11th, and 12th most abundant proteins in EW by mass (Kovacs-Nolan et al., 2005). The levels observed were relatively consistent between egg producers which suggests that the abundances reported here are likely to be generally reflective of those found in chicken eggs. Although the 2D-PAGE and MS analyses of previous workers (Guérin-Dubiard et al., 2006) indicated that Ex-FABP and Cal-γ are considerably less abundant in EW than α-1-ovoglycoprotein, no concentration values had been assigned to Ex-FABP and Cal-γ in EW, until the results reported herein.

**TABLE 4:** Concentration of lipocalin-like proteins found in EW (three trademarks coming from different providers in the United Kingdom) according to western-blot analysis.

	Ex-FABP		Cal-γ		α-1-ovoglycoprotein	
	g/L	μM	g/L	μM	g/L	μM
Cooperative	0.079	4.1	0.095	4.8	2.976	139.1
Clarence court	0.115	6.0	0.123	6.2	6.497	303.6
Happy egg	0.098	5.1	0.113	5.7	5.480	256.1
Average	0.097	5.1	0.110	5.6	4.984	232.9
SD	±0.018	±1.0	±0.014	±0.7	±1.812	±84.7

*To convert the concentration from g/L to molarity, the theoretical MW of the mature proteins was used: 19.1, 19.9, and 21.4 kDa for Ex-FABP, Cal-γ and α1-ovoglycoprotein, respectively.*

The level of α-1-ovoglycoprotein in EW was previously estimated as ∼1 g L^−1^ (Ketterer, 1965), whereas here a considerably higher concentration of 232.9 μM (or 5 g L^−1^) is reported (Table 4). This difference may reflect the use of alcohol precipitation before quantification in the previous report (Ketterer, 1965), which could lead to incomplete protein recovery. Since in the work reported here quantification was achieved directly on EW, it seems reasonable to suggest that the estimation obtained is closer to the true value but might still represent an underestimate as the glycosylation of the native EW protein might reduce its immunoreactivity against the antibodies raised to the non-glycosylated form. Given the previous classification of EW proteins based on abundance (Kovacs-Nolan et al., 2005), α-1-ovoglycoprotein belongs to the high concentration group of “major EW protein” set along with ovalbumin (54 g L^−1^), ovotransferrin (12.0 g L^−1^), ovomucoid (11 g L^−1^), globulin (4 g L^−1^), ovomucin (3.5 g L^−1^) lysozyme (3.4 g L^−1^), and ovoinhibitor (1.5 g L^−1^). However, Cal-γ and Ex-FABP, present at 5.6 and 5.1 μM (or 0.11 and 0.10 g L^−1^), would belong to the “minor EW protein” set, which include ovoflavoprotein (0.8 g L^−1^), ovostatin (0.5 g L^−1^), cystatin (0.05 g L^−1^), and avidin (0.05 g L^−1^).

### Ex-FABP Binds Enterobactin With High Affinity and Strong Preference for the Ferrated Form

**FIGURE 2:**
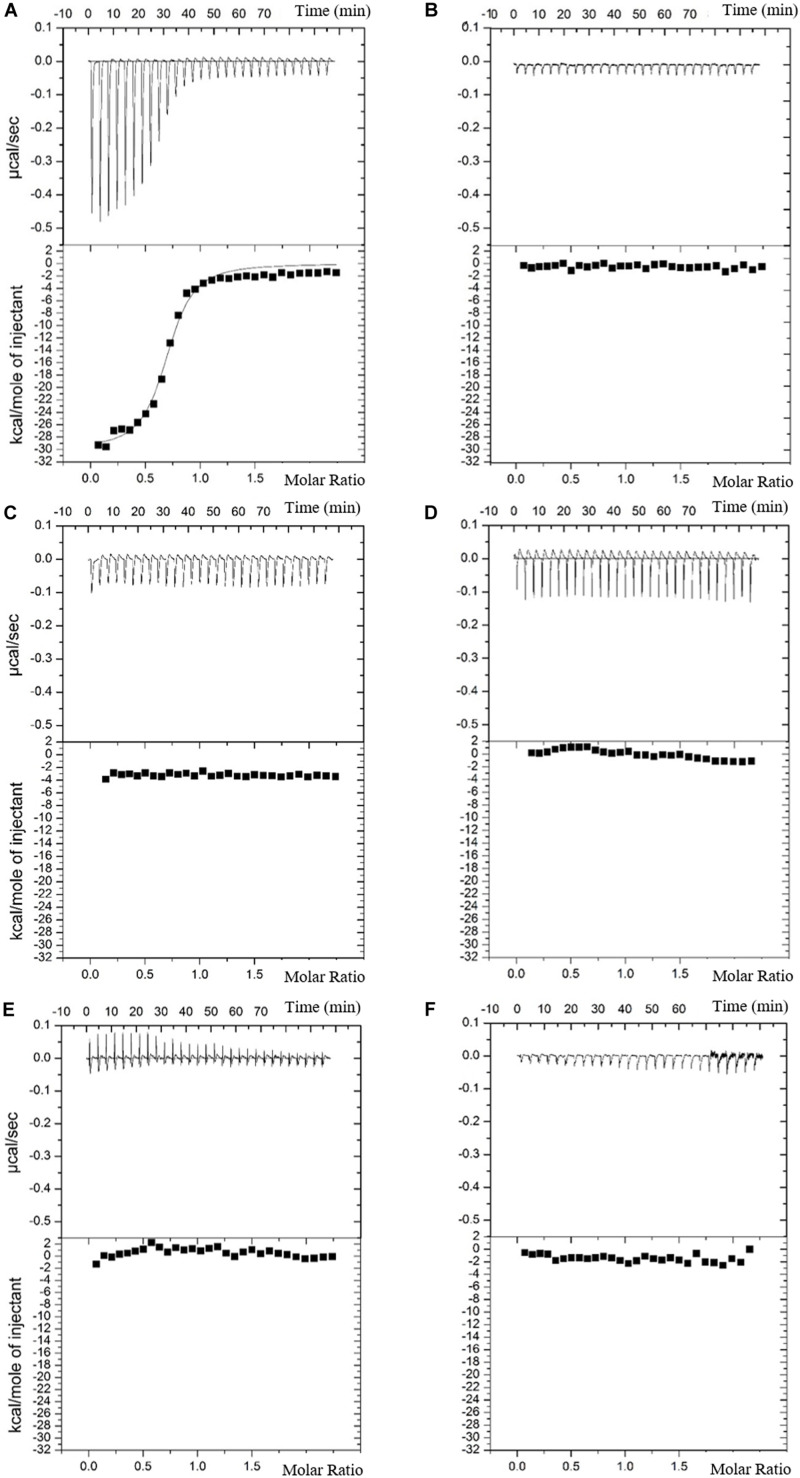
Interactions between lipocalins and *S*E siderophores. Isothermal titration calorimetry was achieved with 29 injections (10 μL) of 50 μM ligands (Ent and Sal) in an adiabatic well containing 5 μM lipocalins. Proteins and ligands were both dissolved in TBS (20 mM Tris, 500 mM NaCl, 1.3% DMSO, pH 7.4) and experiments were achieved at 30°C. No interactions could be measured between Ex-FABP and apo-Sal **(B)**, whereas a K_*d*_ of 86 ± 5 nM was measured between Ex-FABP and apo-Ent **(A)**. No interactions could be measured between Cal-γ and enterobactin **(C)**, Cal-γ and salmochelin **(D)**, α-1-glycoprotein and enterobactin **(E)** and α-1-glycoprotein and salmochelin **(F)**. Data for LCN2, Fe-Ent are shown in the Supplementary Figure S2.

The ability of the three EW lipocalins to specifically bind the iron-free forms of Ent (apoEnt) and Sal (diglucosylated form, apoDGE) was compared with that of LCN2, using isothermal titration calorimetry (ITC). LCN2 bound apoEnt in an exothermic reaction with a high affinity, giving a dissociation constant (K_*d*_) of 58 ± 12 nM (at pH 7.4; Table 5 and Supplementary Figure S4), which is 16-fold higher than the value previously reported using tryptophan quenching fluorescence (TQF; 3.57 nM; Abergel et al., 2006). No binding was observed with apoDGE (Supplementary Figure S4), which matches previous findings showing that LCN2 does not bind DGE (Fischbach et al., 2006). Ex-FABP also bound apoEnt at high affinity, giving an exothermic reaction with a dissociation constant (K_*d*_) of 86 ± 5 nM at pH 7.4 (Table 5 and Figure 2A) and likewise failed to interact with apoDGE, as observed previously (Correnti et al., 2011). It should be noted that the Ex-FABP binding affinity for apoEnt found here is 172-fold weaker than that determined previously (0.5 ± 0.15 nM) using TQF under similar (TSB buffer, pH 7.4) conditions (Correnti et al., 2011). This discrepancy could result from the difference in the techniques used (ITC vs TQF), differences in reaction conditions (e.g. Ex-FABP and DMSO concentrations) or differences in protein preparation. For both LCN2 and Ex-FABP, the apoEnt molar-binding stoichiometry was close to 1:1 at 0.872 and 0.617, respectively (Table 5), which again is consistent with previous reports (Goetz et al., 2002; Correnti et al., 2011).

**TABLE 5:** Lipocalins-binding affinities towards apo- and ferric-ligands obtained using isothermal titration calorimetry (in TBS pH 7.4 at 30°C) in this study and compared values from the literature. K_d_ is the equilibrium dissociation constant, n the binding stoichiometry, ΔS the entropy, and ΔH the enthalpy.

Protein	Ligands	Reference	K_d_ nM	*n*	ΔS cal/mol, K	ΔH cal/mol
LCN2	*apo-Ent*	This study	58.4 ± 12.5	0.872	−58.9	−2.78 × 10^4^
		Abergel et al., 2006	3.57			
	*Fe^3+^-Ent*	This study	11.8 ± 8.8	0.863	−50.2	−2.62 × 10^4^
		Goetz et al., 2002	0.41			
		Fischbach et al., 2006	0.43			
		Li et al., 2015	240			
Ex-FABP	*apo-Sal (or DGE)*	This study	Under threshold			
		Fischbach et al., 2006	Under threshold			
	*apo-Ent*	This study	86.2 ± 14.6	0.617	−60.9	−2.83 × 10^4^
		Correnti et al., 2011	0.5 ± 0.15			
	*Fe^3+^-Ent*	This study	5.3 ± 3.8	0.672	−26.1	−1.94 × 10^4^
		Correnti et al., 2011	0.22 ± 0.06			
	*apo-Sal (or DGE)*	This study	Under threshold			
		Correnti et al., 2011	Under threshold			
Cal-γ	*apo-Ent*	This study	Under threshold			
	*apo-Sal (or DGE)*	This study	Under threshold			
α-1-ovoglycoprotein	*apo-Ent*	This study	Under threshold			
	*apo-Sal (or DGE)*	This study	Under threshold			

Since a previous TQF study indicated that Ex-FABP binds Fe^3+^-Ent with a higher (2.3-fold) affinity than apoEnt (Correnti et al., 2011), and similar findings have been made with LCN2 (8.7-fold; Abergel et al., 2006), the interaction between Fe^3+^-Ent and Ex-FABP was tested here by ITC. The results show a 16-fold higher affinity (K_*d*_ 5.3 ± 3.8 nM) for Fe^3+^-Ent than apoEnt (Table 5 and Supplementary Figure S4) which indicates a higher preference for the ferric- over the apo-form than was previously suggested (Correnti et al., 2011). The ITC data also indicates that LCN2 possesses a 4.9-fold higher affinity (K_*d*_ 11.8 ± 8.8 nM) constant for Fe^3+^-Ent than for apoEnt (Table 5 and Supplementary Figure S2); a similar higher affinity (8.7-fold) was reported by Abergel et al. (2006), and Bao et al. (2010) also showed that Fe^3+^ dramatically enhances the affinity of LCN2 for catechols (by at least 95-fold). It should be noted that the ITC-determined binding affinities of LCN2 for Fe^3+^-Ent reported here (11.8 nM) and previously (240 nM; Li et al., 2015) are both considerably weaker than those determined by TFQ (0.41 and 0.43 nM; Goetz et al., 2002; Fischbach et al., 2006) indicating that differences in published affinity values for LCN2 (and Ex-FABP) are to a large degree related to the methods employed. ITC indicated that neither Cal-γ nor α-1-ovoglycoprotein bind either apoEnt or apoDGE (Table 5 and Figure 2) which suggests that these lipocalins do not act as “siderocalins,” although it remains possible that they could have affinity for siderophores not considered here. It is also important to note that α-1-ovoglycoprotein is glycosylated in EW and such post-translational modifications were not achieved during over-production in *E. coli*. This lack of glycosylation might have influenced the observed bioactivity of the recombinant protein.

### Ex-FABP Inhibits Growth of Salmochelin-Deficient *S*E Mutants in Iron-Restricted Medium When Provided at Concentrations Found in EW

**FIGURE 3:**
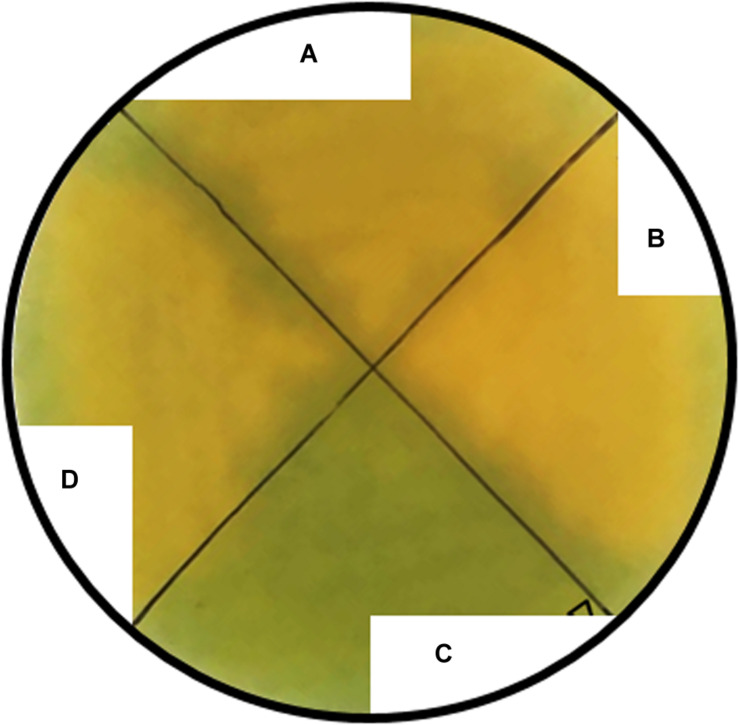
Siderophore production by the wildtype **(A)**, and Δ*iroDEN* **(B)**, Δ*entB* **(C)**, and Δ*iroBC* **(D)** mutants. All strains were grown on CAS plates to determine siderophore secretion capacity. After 12 h of incubation at 37°C, siderophore-producing strains generated a yellow-golden color while for the non-siderophore producing strain the CAS plate remained green.

In order to determine whether EW lipocalins can cause growth inhibition of an *S*E strain producing Ent, but not Sal, a mutant (Δ*iroBC*) lacking the ability to produce and export Sal was generated. The *iroB* gene encodes the glucosyltransferase that converts Ent into Sal, and *iroC* encodes the transporter required for export of Sal across the cytosolic membrane (Hantke et al., 2003; Bister et al., 2004; Crouch et al., 2008); thus, the *iroBC* mutant can neither produce nor release Sal, but should retain the capacity to produce and utilize Ent. Furthermore, two other mutants were generated as controls: Δ*iroDEN* and Δ*entB*. The former should be able to produce both Ent and Sal but was not expected to utilise Sal due to absence of the IroN OM receptor and IroD/IroE esterases (Müller et al., 2009). The latter (Δ*entB*) is unable to convert isochorismate to 2,3-dihydro-2,3-dihydroxybenzoate (an Ent precursor; Gehring et al., 1998) and therefore can produce neither Ent nor its derivative, Sal. The genotype of the mutants was confirmed by genome sequencing and propagation on CAS plates (Louden et al., 2011) showed that, as expected, the Δ*entB* (Ent^–^ Sal^–^) mutant does not produce siderophore whereas both the Δ*iroBC* (Ent^+^ Sal^–^) and Δ*iroDEN* (Ent^+^ Sal^+^) mutants are siderophore producing (Figure 3).

**FIGURE 4:**
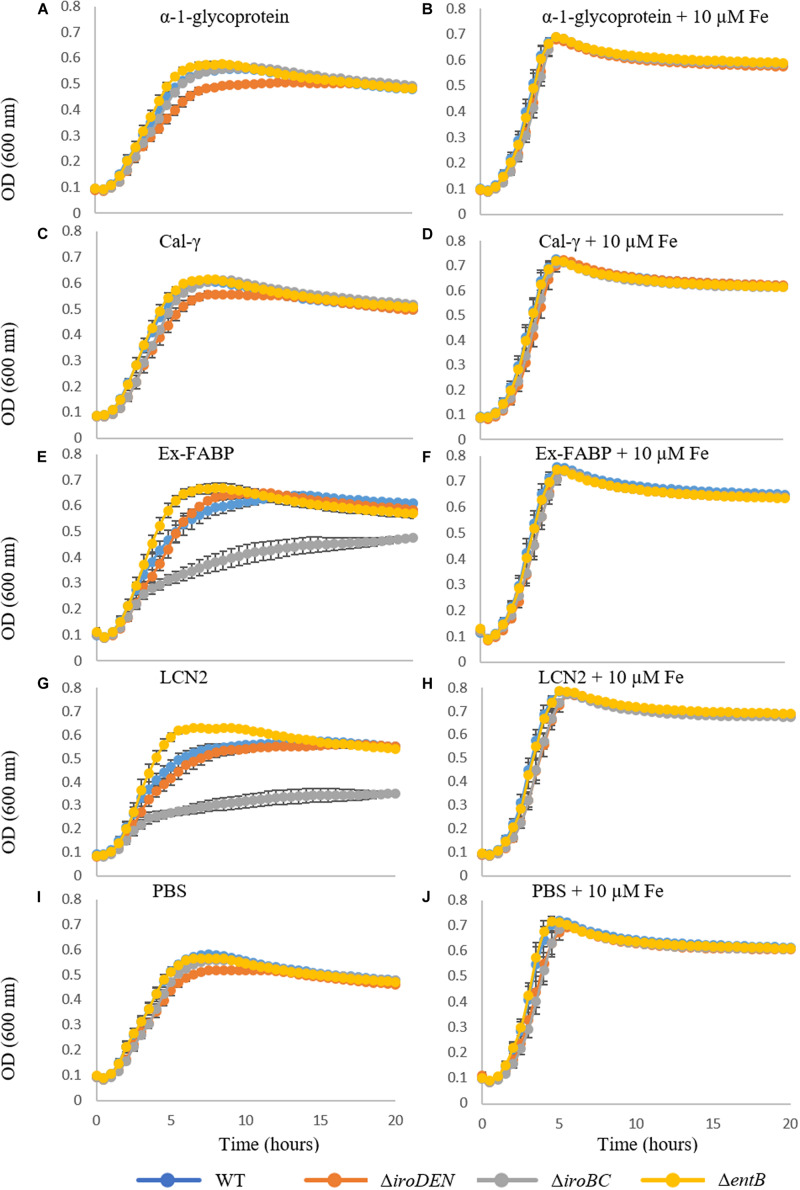
Effect of EW lipocalins on growth of *Salmonella* Enteritidis and mutants defective in siderophore production and/or utilization under iron restriction. Growth of the WT and the Δ*iroBC*, Δ*iroDEN*, and Δ*entB* mutants was compared in M9 medium with (right) or without (left) 10 μM ferric citrate at 37°C and 200 rpm, for 20 h. The medium included 5 μM of either α-1-glycoprotein **(A,B)**, Cal-γ **(C,D)**, Ex-FABP **(E,F)**, LCN2 **(G,H)** or ∼100 μL of PBS **(I,J)**. Error bars indicate standard error from three biological replicates with two technical replicates.

To assess whether exposure to EW lipocalins negatively impacts *S*E growth through Ent sequestration, growth of the siderophore-defective *S*E mutants and wildtype was monitored in the presence and absence of each of the EW lipocalins and LCN2 (at 5 μM), under low- and sufficient-iron conditions (Figure 4). Of the lipocalins tested, only Ex-FABP and LCN2 had any impact on iron-deficient growth (Figure 4), and none had any impact under iron sufficiency. The Δ*iroBC* mutant (Ent^+^ Sal^–^) was particularly affected with a 2.5-fold growth reduction (at 6 h; Supplementary Figure S5) under low-iron with Ex-FABP or LCN2 (Figures 4E,G). This reduced growth is fully consistent with the Ent sequestering activities of Ex-FABP and LCN2, and the dependence of the Δ*iroBC* mutant on Ent as the sole siderophore supporting its low-iron growth. The wildtype also displayed a reduced growth with Ex-FABP or LCN2 under low iron, but this was far more modest (at just 1.25-fold; at 6 h; Supplementary Figure S5) than that seen for the Δ*iroBC* mutant. This mild reduction in iron-restricted growth is consistent with the co-dependence of the wildtype on Ent and Sal under iron deficiency. Thus, production of Sal by the wildtype appears to largely overcome the impact of Fe-Ent sequestration by Ex-FABP or LCN2, as reported previously for other bacteria (Fischbach et al., 2006; Correnti et al., 2011).

In contrast to the Δ*iroBC* mutant, the Δ*entB* mutant (Ent^–^ Sal^–^) exhibited better low-iron growth (1.2-fold at 6 h; Supplementary Figure S5) than the wildtype in the presence of Ex-FABP or LCN2 (Figures 4E,G), with no apparent reduction in growth exhibited in the presence of these lipocalins. The failure of Ex-FABP or LCN2 to inhibit the Δ*entB* mutant is consistent with the ability of this mutant to escape competition for iron by LCN2 or Ex-FABP due its lack of Ent production. Thus, the results indicate that production of Ent as sole siderophore is deleterious to growth under low-iron conditions in the presence of Ex-FABP or LCN2. The Δ*iroDEN* mutant showed reduced growth with respect to the other three strains (∼1.1-fold at 6 h) under low-iron conditions in the absence of Ex-FABP or LCN2 (Figure 4I). This indicates that the capacity to secrete Sal, but not utilize it, results in deficient growth under iron restriction, which presumably arises due to sequestration of extracellular iron by secreted Sal. Cal-γ and α-1-ovoglycoprotein had no apparent effect on the growth of *S*E or its mutants under low- or sufficient-iron conditions (Figure 4). This is consistent with the ITC data which indicate that these lipocalins do not bind Ent or Sal.

In summary, Ex-FABP at 5 μM caused a major inhibition of iron-restricted growth for an *S*E strain relying on Ent as sole siderophore but had only a modest impact on the wildtype deploying Sal alongside Ent. Since the western blotting data indicate that Ex-FABP is present in EW at 5.1 μM, these results suggest that Ex-FABP concentrations in EW are sufficient to cause inhibition of iron-restricted growth for an *S*E strain, and potentially other bacterial species, relying on Ent as sole siderophore.

**FIGURE 5:**
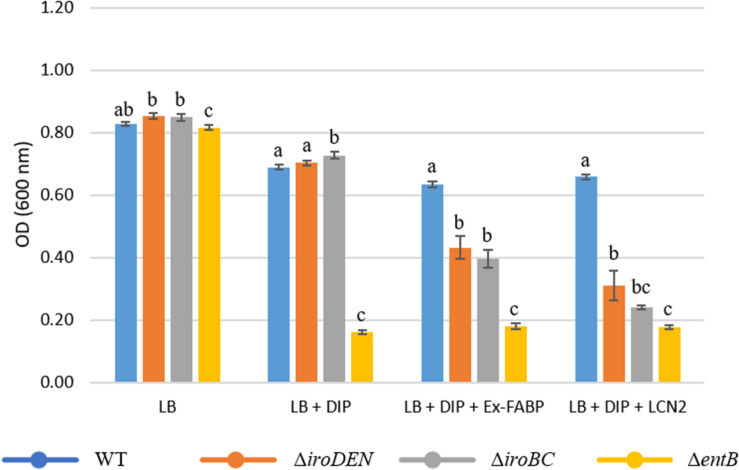
Effect of Ex-FABP on growth of *Salmonella* Enteritidis and mutants defective in siderophore production and/or utilization under iron restriction in rich medium (LB broth). Growth was at 37°C with shaking and the OD values presented are those achieved at 20 h of growth. The chelator 2,2′-dipyridyl (DIP) was at 200 μM where indicated, and Ex-FABP or LCN2 were present at 5 μM (where indicated). The error bars are the standard error calculated from three biological replicates with three technical replicates. For each growth medium, one-way ANOVA followed by a multiple comparison of means (Tukey Contrasts) was achieved using R software (version 3.5.3). This allowed to identify groups significantly different (identified as “a”, “b”, and “c”).

Although no iron-restricted growth defect was observed for the Δ*entB* mutant in minimal (M9) medium, when growth tests were performed in rich (LB) medium with the iron chelator DIP, a major reduction in growth (∼4-fold), with respect to the wildtype, was observed (Figure 5). No growth defect was seen in the absence of DIP indicating that the phenotype obtained is related to iron restriction imposed by DIP chelation. This observation matches previous work (Liu et al., 2015) and likely arises from the inability of the Δ*entB* mutant to effectively compete with DIP for iron due to its lack of Ent (and Sal) production. In contrast to the Δ*entB* mutant, the Δ*iroDEN* and Δ*iroBC* strains showed no growth defect in LB with DIP (Figure 5), presumably because they retain the ability to produce and utilize Ent, and can thus compete with DIP for iron.

As expected, the provision of either Ex-FABP or LCN2 had little impact on the weak growth of the Δ*entB* mutant in LB with DIP (Figure 5). However, the Δ*iroDEN* and Δ*iroBC* mutants showed a significant growth defect (∼1.5–2-fold reduction) compared to the wildtype in LB with DIP when 5 μM Ex-FABP or LCN2 were provided (Figure 5). This is consistent with the ability of Ex-FABP and LCN2 to sequester Ent, but not Sal. LCN2 caused a greater growth reduction than Ex-FABP (2-fold cf. 1.5-fold, respectively) for the Δ*iroDEN* and Δ*iroBC* mutants, and the growth of Δ*iroBC* mutant was slightly more reduced than that of the Δ*iroDEN* mutant (Figure 5).

The findings above are fully consistent with the Ent-sequestering roles of LCN2 and Ex-FABP, as shown here and previously (Goetz et al., 2002; Fischbach et al., 2006; Correnti et al., 2011). The results above are also in accordance with previous work showing that mutations in *iroBC* (Sal production and export) and *iroN* (Sal uptake) do not impair growth of *S.* Typhimurium in rich (LB) medium (Raffatellu et al., 2009) but do significantly reduce growth in (low iron) tissue culture medium (DMEM) containing LCN2. The present study also shows that Ex-FABP is present at sufficient concentration in EW to cause inhibition of iron-restricted growth for an *S*E strain relying on Ent as sole siderophore, but not for *S*E deploying Sal alongside Ent. These results reflect previous findings showing that 2.5 and 10 μM Ex-FABP cause iron-restricted growth inhibition of Ent-producing *E. coli* (Correnti et al., 2011; Garénaux et al., 2013) and that complementation with *iroBCDEN* reverses inhibition by Ex-FABP (Garénaux et al., 2013).

## Conclusion

The results presented here show for the first time that *S*E, the major pathogen associated with egg-linked food-borne outbreaks, is resistant to the enhanced iron-restriction imposed by the EW-lipocalin, Ex-FABP. This resistance is due to the ability of *S*E to produce and utilize the siderophore, Sal. This Sal-resistance property would be expected to contribute to survival of *S*E in the iron-restricted conditions of EW and would thus be likely to support the capacity of *S*E to infect eggs (Clavijo et al., 2006; Gantois et al., 2009; Vylder et al., 2013; Baron et al., 2016). Furthermore, the results provide the first immuno-quantification of the three EW lipocalins in eggs and reveal that Ex-FABP is present at concentrations (5.1 μM) sufficient to inhibit iron-restricted, Ent-dependent growth. In contrast, neither Cal-γ nor α-1-ovoglycoprotein influenced the growth of *S*E under the conditions tested here, indicating that these two EW lipocalins do not bind the siderophores produced by *S*E and do not contribute directly to iron restriction; the ITC data fully support the lack of Ent or Sal binding activity by these lipocalins. ITC also showed that both Ex-FABP and LCN2 bind Ent (but not Sal) with high affinity and with a preference for Fe-Ent (Kd of 5.4 and 11.8 nM, respectively) over apo-Ent (Kd of 86.2 and 58.4 nM, respectively). These binding patterns are consistent with previous work (Goetz et al., 2002; Abergel et al., 2006; Fischbach et al., 2006; Bao et al., 2010; Correnti et al., 2011; Li et al., 2015) although the affinity value ranges reported here and elsewhere show wide variation, which largely correlate with the methodology (ITC or TFQ) employed. A key finding of the results provided here is that the natural concentration of Ex-FABP in EW appears sufficient to play a biological role in limiting bacterial growth through sequestration of the siderophore, Ent. However, this effect is overcome by *S*E through its ability to deploy a Sal as second, “stealth” siderophore.

## Data Availability Statement

All datasets generated for this study are included in the article/Supplementary Material.

## Author Contributions

LJ, FB, SB, SJ, and SA designed the experiments. LJ and CF performed the experiments and analyzed the corresponding results. LJ wrote the manuscript with FB, SB, CG-D, FN, MG, KK, SJ, and SA.

## Conflict of Interest

The authors declare that the research was conducted in the absence of any commercial or financial relationships that could be construed as a potential conflict of interest.

## Footnotes

1 https://www.thermofisher.com/fr/fr/home/life-science/cloning/gene-synthesis.html

## References

[B1] Abergel R. J. Moore E. G. Strong R. K. Raymond K. N. (2006). Microbial evasion of the immune system: structural modifications of enterobactin impair siderocalin recognition. *J. Am. Chem. Soc.* 128 10998–10999. 10.1021/ja062476+ 16925397 PMC3188317

[B2] Alderton G. Ward W. H. Fevold H. L. (1946). Identification of the bacteria-inhibiting iron-binding protein of egg-white as conalbumin. *Arch. Biochem. Biophys.* 11 9–13. 20998021

[B3] Andrews S. C. Robinson A. K. Rodríguez-Quiñones F. (2003). Bacterial iron homeostasis. *FEMS Microbiol. Rev.* 27 215–237. 12829269 10.1016/S0168-6445(03)00055-X

[B4] Bao G. Clifton M. Hoette T. M. Mori K. Deng S. X. Qiu A. (2010). Iron traffics in circulation bound to a siderocalin (Ngal)-catechol complex. *Nat. Chem. Biol.* 6 602–609. 10.1038/nchembio.402 20581821 PMC2907470

[B5] Baron F. Gautier M. Brulé G. (1997). Factors involved in the inhibition of growth of *Salmonella enteritidis* in liquid egg white. *J. Food Prot.* 60 1318–1323. 10.4315/0362-028X-60.11.1318 31207765

[B6] Baron F. Nau F. Guérin-Dubiard C. Bonnassie S. Gautier M. Andrews S. C. (2016). Egg white versus *Salmonella* Enteritidis! A harsh medium meets a resilient pathogen. *Food Microbiol.* 53 82–93. 10.1016/j.fm.2015.09.009 26678134

[B7] Bäumler A. J. Norris T. L. Lasco T. Voigt W. Reissbrodt R. Rabsch W. (1998). IroN, a novel outer membrane siderophore receptor characteristic of *Salmonella enterica*. *J. Bacteriol.* 180 1446–1453. 9515912 10.1128/jb.180.6.1446-1453.1998 PMC107043

[B8] Bister B. Bischoff D. Nicholson G. J. Valdebenito M. Schneider K. Winkelmann G. (2004). The structure of salmochelins: C-glucosylated enterobactins of *Salmonella enterica*. *Biometals* 17 471–481. 10.1023/b:biom.0000029432.69418.6a 15259369

[B9] Bullen J. J. Rogers H. J. Griffiths E. (1978). Role of iron in bacterial infection. *Curr. Top. Microbiol. Immunol.* 80 1–35. 352628 10.1007/978-3-642-66956-9_1

[B10] Carrano C. J. Raymond K. N. (1979). Ferric ion sequestering agents 2. Kinetics and mechanism of iron removal from transferrin by enterobactin and synthetic tricatechols. *J. Am. Chem. Soc.* 101 5401–5404.

[B11] Chart H. Rowe B. (1993). Iron restriction and the growth of *Salmonella enteritidis*. *Epidemiol. Infect.* 110 41–47. 8432322 10.1017/s0950268800050664 PMC2271952

[B12] Cherepanov P. P. Wackernagel W. (1995). Gene disruption in *Escherichia coli*: TcR and KmR cassettes with the option of Flp-catalyzed excision of the antibiotic-resistance determinant. *Gene* 158 9–14. 10.1016/0378-1119(95)00193-a 7789817

[B13] Clavijo R. I. Loui C. Andersen G. L. Riley L. W. Lu S. (2006). Identification of genes associated with survival of *Salmonella enterica* serovar Enteritidis in chicken egg albumen. *Appl. Environ. Microbiol.* 72 1055–1064. 10.1128/AEM.72.2.1055-1064.2006 16461649 PMC1392908

[B14] Clifton M. C. Corrent C. Strong R. K. (2009). Siderocalins: siderophore-binding proteins of the innate immune system. *Biometals* 22 557–564. 10.1007/s10534-009-9207-6 19184458

[B15] Correnti C. Clifton M. C. Abergel R. J. Allred B. Hoette T. M. Ruiz M. (2011). Galline Ex-FABP is an antibacterial siderocalin and a lysophosphatidic acid sensor functioning through dual ligand specificities. *Structure* 19 1796–1806. 10.1016/j.str.2011.09.019 22153502 PMC3240821

[B16] Crouch M. V. Castor M. Karlinsey J. E. Kalhorn T. Fang F. C. (2008). Biosynthesis and IroC-dependent export of the siderophore salmochelin are essential for virulence of *Salmonella enterica* serovar Typhimurium. *Mol. Microbiol.* 67 971–998. 10.1111/j.1365-2958.2007.06089.x 18194158

[B17] D’Ambrosio C. Arena S. Scaloni A. Guerrier L. Boschetti E. Mendieta M. E. (2008). Exploring the chicken egg white proteome with combinatorial peptide ligand libraries. *J. Proteome Res.* 7 3461–3474. 10.1021/pr800193y 18570458

[B18] Datsenko K. A. Wanner B. L. (2000). One-step inactivation of chromosomal genes in *Escherichia coli* K-12 using PCR products. *Proc. Natl. Acad. Sci. U.S.A.* 97 6640–6645. 10.1073/pnas.120163297 10829079 PMC18686

[B19] ECDC and EFSA (2017). *Multicountry Outbreak of Salmonella Enteritidis Phage Type 8, MLVA type 2-9-7-3-2 and 2-9-6-3-2 Infections.* Stockholm and Parma: ECDC and EFSA.

[B20] Efsa Panel on Biological Hazards. (2014). Scientific Opinion on the public health risks of table eggs due to deterioration and development of pathogens. *EFSA J.* 12 1–147.

[B21] Efsa Panel on Biological Hazards. (2019). Scientific opinion on the *Salmonella* control in poultry flocks and its public health impact. *EFSA J.* 17 1–155. 10.2903/j.efsa.2019.5596 PMC7009056 32626222

[B22] Fischbach M. A. Lin H. Zhou L. Yu Y. Abergel R. J. Liu D. R. (2006). The pathogen-associated iroA gene cluster mediates bacterial evasion of lipocalin 2. *Proc. Natl. Acad. Sci. U.S.A.* 103 16502–16507. 10.1073/pnas.0604636103 17060628 PMC1637611

[B23] Ford S. Cooper R. A. Evans R. W. Hider R. C. Williams P. H. (1988). Domain preference in iron removal from human transferrin by the bacterial siderophores aerobactin and enterochelin. *Eur. J. Biochem.* 178 477–481. 10.1111/j.1432-1033.1988.tb14473.x 2974803

[B24] Gantois I. Ducatelle R. Pasmans F. Haesebrouck F. Gast R. Humphrey T. J. (2009). Mechanisms of egg contamination by *Salmonella* Enteritidis: review article. *FEMS Microbiol. Rev.* 33 718–738. 19207743 10.1111/j.1574-6976.2008.00161.x

[B25] Garénaux A. Houle S. Folch B. Dallaire G. Truesdell M. Lépine F. (2013). Avian lipocalin expression in chickens following *Escherichia coli* infection and inhibition of avian pathogenic *Escherichia coli* growth by Ex-FABP. *Vet. Immunol. Immunopathol.* 152 156–167. 10.1016/j.vetimm.2012.09.018 23102565

[B26] Garibaldi J. A. (1970). Role of microbial iron transport compounds in bacterial spoilage of eggs. *Appl. Microbiol.* 20 558–560. 5498603 10.1128/am.20.4.558-560.1970 PMC376988

[B27] Gast R. K. Beard C. W. (1990). Production of *Salmonella enteritis*-contaminated eggs by experimentally infected hens. *Avian. Dis.* 34 438–446. 2196046

[B28] Gehring A. M. Mori I. Walsh C. T. (1998). Reconstitution and characterization of the *Escherichia coli* enterobactin synthetase from EntB, EntE, and EntF. *Biochemistry* 37 2648–2659. 10.1021/bi9726584 9485415

[B29] Goetz D. H. Holmes M. A. Borregaard N. Bluhm M. E. Raymond K. N. Strong R. K. (2002). The neutrophil lipocalin NGAL is a bacteriostatic agent that interferes with siderophore-mediated iron acquisition. *Mol. Cell.* 10 1033–1043. 10.1016/s1097-2765(02)00708-6 12453412

[B30] Guérin-Dubiard C. Pasco M. Mollé D. Désert C. Croguennec T. Nau F. M. (2006). Proteomic analysis of hen egg white. *J. Agric. Food Chem.* 54 3901–3910. 10.3382/ps.2012-02986 16719513

[B31] Haginaka J. Seyama C. Kanasugi N. (1995). Ovoglycoprotein-Bonded HPLC Stationary Phases for Chiral Recognition. *Anal. Chem.* 67 2539–2547. 10.1021/ac00111a008 8849023

[B32] Hantke K. Nicholson G. Rabsch W. Winkelmann G. (2003). Salmochelins, siderophores of *Salmonella enterica* and uropathogenic *Escherichia coli* strains, are recognized by the outer membrane receptor IroN. *Proc. Natl. Acad. Sci. U.S.A.* 100 3677–3682. 10.1073/pnas.0737682100 12655053 PMC152981

[B33] Holmes M. A. Paulsene W. Jide X. Ratledge C. Strong R. K. (2005). Siderocalin (Lcn 2) also binds carboxymycobactins, potentially defending against mycobacterial infections through iron sequestration. *Structure* 13 29–41. 10.1016/j.str.2004.10.009 15642259

[B34] Humphrey T. J. Whitehead A. Gawler A. H. L. Henley A. (1991). Numbers of *Salmonella enteritidis* in the contents of naturally contaminated hens’ eggs. *Epidemiol. Infect.* 106 489–496. 10.1017/s0950268800067546 2050203 PMC2271858

[B35] Johnstone T. C. Nolan E. M. (2015). Beyond iron: non-classical biological functions of bacterial siderophores. *Dalton trans.* 44 6320–6339. 10.1039/c4dt03559c 25764171 PMC4375017

[B36] Julien L. A. Baron F. Bonnassie S. Nau F. Guérin C. Jan S. (2019). The anti-bacterial iron-restriction defence mechanisms of egg white; the potential role of three lipocalins in resistance against *Salmonella*. *Biometals* 32 453–467. 10.1007/s10534-019-00180-w 30810876 PMC6584246

[B37] Keller L. H. Benson C. E. Krotec K. Eckroade R. J. (1995). *Salmonella* Enteritidis colonization of the reproductive tract and forming and freshly laid eggs of chickens. *Infect. Immun.* 63 2443–2449. 7790055 10.1128/iai.63.7.2443-2449.1995 PMC173326

[B38] Kelly A. U. McSorley S. T. Patel P. Talwar D. (2017). Interpreting iron studies. *BMJ* 357 1–6. 10.1136/bmj.j2513 28620083

[B39] Ketterer B. B. (1965). Ovoglycoprotein, a protein of hen egg white. *Biochem. J.* 96 372–376. 5837783 10.1042/bj0960372 PMC1207049

[B40] Kovacs-Nolan J. Phillips M. Mine Y. (2005). Advances in the value of eggs and egg components for human health. *J. Agric. Food Chem.* 53 8421–8431. 10.1021/jf050964f 16248532

[B41] Li W. Cui T. Hu L. Wang Z. Li Z. He Z. G. (2015). Cyclic diguanylate monophosphate directly binds to human siderocalin and inhibits its antibacterial activity. *Nat. Commun.* 6 1–9. 10.1038/ncomms9330 PMC4595737 26390966

[B42] Liu Y. Zhang Q. Hu M. Yu K. Fu J. Zhou F. (2015). Proteomic analyses of intracellular *Salmonella enterica* serovar typhimurium reveal extensive bacterial adaptations to infected host epithelial cells. *Infect. Immun.* 83 2897–2906. 10.1128/IAI.02882-14 25939512 PMC4468536

[B43] Lock J. L. Board R. G. (1992). Persistence of contamination of hens’ egg albumen in vitro with *Salmonella* serotypes. *Epidemiol. Infect.* 108 389–396. 10.1017/s095026880004989x 1601073 PMC2272201

[B44] Louden B. C. Haarmann D. Lynne A. M. (2011). Use of blue agar CAS assay for siderophore detection. *J. Microbiol. Biol. Educ.* 12 51–53. 10.1128/jmbe.v12i1.249 23653742 PMC3577196

[B45] Luo M. Lin H. Fischbach M. A. Liu D. R. Walsh C. T. Groves J. T. (2006). Enzymatic tailoring of enterobactin alters membrane partitioning and iron acquisition. *ACS Chem. Biol.* 1 29–32. 10.1021/cb0500034 17163637

[B46] Mahmood T. Yang P. C. (2012). Western blot: technique, theory, and trouble shooting. *N. Am. J. Med. Sci.* 4 429–434. 10.4103/1947-2714.128482 23050259 PMC3456489

[B47] Mann K. (2007). The chicken egg white proteome. *Proteomics* 7 3558–3568. 17722208 10.1002/pmic.200700397

[B48] Mann K. Mann M. (2011). In-depth analysis of the chicken egg white proteome using an LTQ Orbitrap Velos. *Proteome Sci.* 9 1–6. 10.1186/1477-5956-9-7 21299891 PMC3041730

[B49] Müller S. I. Valdebenito M. Hantke K. (2009). Salmochelin, the long-overlooked catecholate siderophore of *Salmonella*. *BioMetals* 22 691–695. 10.1007/s10534-009-9217-4 19214756

[B50] Nys Y. Sauveur B. (2004). Valeur nutritionnelle des oeufs, INRA. *Prod. Anim.* 5 385–393.

[B51] Pollack J. R. Ames B. N. Neilands J. B. (1970). Iron transport in *Salmonella typhimurium*: mutants blocked in the biosynthesis of enterobactin. *J. Bacteriol.* 104 635–639. 4923066 10.1128/jb.104.2.635-639.1970 PMC285038

[B52] Raffatellu M. George M. D. Akiyama Y. Hornsby M. J. Nuccio S. P. Paixao T. A. (2009). Lipocalin-2 resistance confers an advantage to *Salmonella enterica* serotype typhimurium for growth and survival in the inflamed intestine. *Cell Host Microbe* 5 476–486. 10.1016/j.chom.2009.03.011 19454351 PMC2768556

[B53] Sambrook J. Russell D. W. (2001). *Molecular Cloning: A Laboratory Manual.* Cold Spring Harbor: Cold Spring Harbor Laboratory Press.

[B54] Schade A. Caroline L. (1944). Raw hen egg white and therole of iron in growth inhibition of *Shigella* dysenteriae, *Staphylococcus aureus*, *Escherichia coli* and *Saccharomyces cerevisiae*. *Science* 100 14–15. 10.1126/science.100.2584.14 17783793

[B55] USDA (2010). *National Nutrient Database for Standard Reference.* Washington, DC: USDA.

[B56] Valdebenito M. Müller S. I. Hantke K. (2007). Special conditions allow binding of the siderophore salmochelin to siderocalin (NGAL-lipocalin). *FEMS Microbiol. Lett.* 277 182–187. 10.1111/j.1574-6968.2007.00956.x 18031338

[B57] Vylder J. De, Raspoet R. Dewulf J. Haesebrouck F. Ducatelle R. (2013). *Salmonella* Enteritidis is superior in egg white survival compared with other *Salmonella* serotypes. *Poult. Sci. Assoc. Inc.* 92 842–845. 10.3382/ps.2012-02668 23436537

[B58] Watts R. E. Totsika M. Challinor V. L. Mabbett A. N. Ulett G. C. Voss J. J. (2012). Contribution of siderophore systems to growth and urinary tract colonization of asymptomatic bacteriuria *Escherichia coli*. *Infect. Immun.* 80 333–344. 10.1128/IAI.05594-11 21930757 PMC3255690

[B59] Williams J. (1968). A comparison of glycopeptides from the ovotransferrin and serum transferrin of the hen. *Biochem. J.* 108 57–67. 10.1042/bj1080057 5690539 PMC1198769

