# Supplementary material for: The Three Lipocalins of Egg-White: Only Ex-FABP Inhibits Siderophore-Dependent Iron Sequestration by Salmonella Enteritidis
Source: Front Microbiol. 2020 May 15;11:913. doi: 10.3389/fmicb.2020.00913 (PMC7242566; doi:10.3389/fmicb.2020.00913)
Supplement: Supplementary file 1 [file Data_Sheet_1.docx]

Supplementary Material

# Supplementary Figures


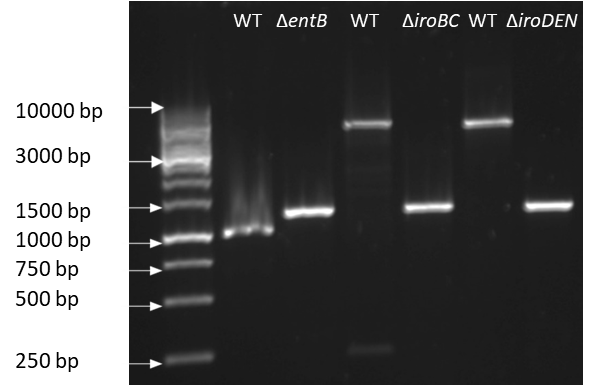


**Supplementary Figure 1.** Electrophoretic analysis of DNA fragments resulting from PCR amplification of genomic DNA isolated from chloramphenicol resistant *S*E strains or wild type *S*E (for primers used, see supplementary Table 1). DNA fragments were subject to electrophoresis in a 0.7% agarose gel in 0.5X TBE. For every mutant, fragments of 1400 bp, matching the size of the chloramphenicol cassette and flanking region were successfully amplified (for expected amplicon sizes, see supplementary Table 1). This suggests that the cassette has been inserted at the right location in the genome, replacing the targeted gene found in the WT in each case.

1. LCN2 sequence once cloned into pET21a:

5’AGGAGATATACATATGCAGGATAGCACCAGCGATCTGATTCCGGCACCGCCTCTGAGCAAAGTTCCGCTGCAGCAGAATTTTCAGGATAATCAGTTTCAAGGCAAGTGGTATGTTGTTGGTCTGGCAGGTAATGCAATTCTGCGTGAAGATAAAGATCCGCAGAAAATGTATGCCACCATCTATGAACTGAAAGAGGACAAAAGCTATAACGTTACCAGCGTTCTGTTTCGCAAAAAAAAGTGCGATTATTGGATCCGTACCTTTGTTCCGGGTTGTCAGCCTGGTGAATTTACCCTGGGTAACATTAAAAGCTATCCGGGTCTGACCAGCTATCTGGTTCGTGTTGTTAGCACCAATTATAACCAGCATGCCATGGTGTTCTTCAAAAAAGTTAGCCAGAATCGCGAGTACTTCAAAATTACCCTGTATGGTCGTACCAAAGAACTGACCAGCGAGCTGAAAGAAAACTTTATTCGTTTTAGCAAAAGCCTGGGTCTGCCGGAAAATCATATTGTGTTTCCGGTTCCGATTGATCAGTGTATTGATGGCCTCGAGCACCACCACCACCACCACTGA**3’**

Translated sequence:

MQDSTSDLIPAPPLSKVPLQQNFQDNQFQGKWYVVGLAGNAILREDKDPQKMYATIYELKEDKSYNVTSVLFRKKKCDYWIRTFVPGCQPGEFTLGNIKSYPGLTSYLVRVVSTNYNQHAMVFFKKVSQNREYFKITLYGRTKELTSELKENFIRFSKSLGLPENHIVFPVPIDQCIDGLEHHHHHH*

1. Ex-FABP sequence once cloned into pET21a:

5’AGGAGATATACATATGAAATACCTGCTGCCGACCGCTGCTGCTGGTCTGCTGCTCCTCGCTGCCCAGCCGGCGATGGCCGCAGCAACAGTTCCGGATCGTAGCGAAGTTGCAGGTAAATGGTATATTGTTGCACTGGCAAGCAACACCGATTTTTTTCTGCGTGAAAAAGGCAAGATGAAGATGGTTATGGCACGTATTAGCTTTCTGGGTGAAGATGAACTGGAAGTTAGCTATGCAGCACCGAGTCCGAAAGGTTGTCGTAAATGGGAAACCACCTTCAAAAAAACCAGTGATGATGGCGAACTGTATTATAGCGAAGAAGCCGAAAAAACCGTTGAAGTTCTGGATACCGACTATAAAAGCTATGCCGTTATTTTTGCGACCCGTGTTAAAGATGGTCGTACCCTGCACATGATGCGTCTGTATAGCCGTAGCCGTGAAGTTAGCCCGACCGCAATGGCAATTTTTCGTAAACTGGCACGTGAACGCAATTATACCGATGAAATGGTTGCAGTTCTGCCGAGCCAAGAGGAATGTAGCGTTGATGAAGTTCTCGAG CACCACCACCACCACCACTGA**3’**

Translated sequence:

 MKYLLPTAAAGLLLLAAQPAMAAATVPDRSEVAGKWYIVALASNTDFFLREKGKMKMVMARISFLGEDELEVSYAAPSPKGCRKWETTFKKTSDDGELYYSEEAEKTVEVLDTDYKSYAVIFATRVKDGRTLHMMRLYSRSREVSPTAMAIFRKLARERNYTDEMVAVLPSQEECSVDEVLEHHHHHH*

1. α-1-glycoprotein sequence once cloned into pET21a:

5’AGGAGATATACATATGAAATACCTGCTGCCGACCGCTGCTGCTGGTCTGCTGCTCCTCGCTGCCCAGCCGGCGATGGCCACCGAAAGTCCGACATGTGCACCGCTGGTTCCGGCAGATATGGATAATGCAACCGTTGATCGTCTGTTAGGTCATTGGGTGTATATTATGGGTGCAAGCCAGTATCCGCCTCACATGGCAGAAATGCGTGAACTGAAATATGCAACCTTTACACTGTTTCCGGGTAGCCATGAAGATGAATTTAATGTGACCGAAATTATGCGCCTGAATGAAACCTGTGTTGTGAAAAACAGCAGCAAAATTCATGTGTTTCGCCATAATAGCACCCTGACACACGAAGATGGTCAGGTTGTTAGCATGGCCGAACTGATTCATAGCGATAAAGACCTGTTTATCCTGAAGCACTTCAAAGATAATCATGTTGGTCTGAGCCTGAGCGCACGTACCGCAGAAGTTACCAAAGAACAGCTGGAAGAATTTGAAGCACAGCTGCGTTGTCATGGTTTTAAACTGGAAGAAGCCTTTATTACGAGCCCGAAAGATGCATGTCCGGCAGCCGGTGAAGAAACCGGTGAAGGTAGCGCAGCAACAGCAGAACCGCAGCTGGGCCTCGAGCACCACCACCACCACCACTGA3’

Translated sequence:

MKYLLPTAAAGLLLLAAQPAMATESPTCAPLVPADMDNATVDRLLGHWVYIMGASQYPPHMAEMRELKYATFTLFPGSHEDEFNVTEIMRLNETCVVKNSSKIHVFRHNSTLTHEDGQVVSMAELIHSDKDLFILKHFKDNHVGLSLSARTAEVTKEQLEEFEAQLRCHGFKLEEAFITSPKDACPAAGEETGEGSAATAEPQLGLEHHHHHH*

1. Cal gamma sequence once cloned into pET21a:

5’AGGAGATATACATATGAAATACCTGCTGCCGACCGCTGCTGCTGGTCTGCTGCTCCTCGCTGCCCAGCCGGCGATGGCCAATAGCATTCCGGTTCAGGCAGATTTTCAGCAGGATAAACTGGCAGGTCGTTGGTATAGCATTGGTCTGGCAAGCAATAGCAACTGGTTCAAAGATAAAAAGCATCTGCTGAAGATGTGCACCACCGATATTGCAGTTACCGCAGATGGTAATATGGAAGTTACCAGCACCTATCCGAAAGGTGAACAGTGTGAAAAACGTAACAGCCTGTATATTCGTACCGAACAGCCTGGTCGTTTTAGTTATACCAATCCGCGTTGGGGTAGCAATCATGATATTCGTGTTGTGGAAACCAACTATGATGAATATGCACTGGTTGCGACCCAGATTAGCAAAAGCACCGGTAGCAGCAATATGGTTCTGCTGTATAGCCGTACCAAAGAAGTTGCACCGCAGCGTCTGGAACGTTTTATGCAGTTTAGCCAAGAACAGGGTCTGAAAGATGAAGAAATTCTGATTCTGCCGCAGACCGATAAATGTATGGCAGATGCAGCACTCGAGCACCACCACCACCACCACTGA**3’**

Translated sequence:

MKYLLPTAAAGLLLLAAQPAMANSIPVQADFQQDKLAGRWYSIGLASNSNWFKDKKHLLKMCTTDIAVTADGNMEVTSTYPKGEQCEKRNSLYIRTEQPGRFSYTNPRWGSNHDIRVVETNYDEYALVATQISKSTGSSNMVLLYSRTKEVAPQRLERFMQFSQEQGLKDEEILILPQTDKCMADAALEHHHHHH*

**Supplementary Figure 2.** Codon optimised nucleotide sequences and corresponding translation sequences of lipocalin genes in overexpression plasmids: pET-lcn2 (A); pET-Ex-FABP (B); pET-Cal-γ (C); and pET-α1-glyc (D). The plasmid sequences are underscored; *Nde*I and *Xho*I sites are in yellow highlight; *pelB* sequence is in green highlight. The translation product amino acid sequence shows the ‘additional’ residues (not present in the native protein) highlighted: blue, Leu-Glu peptide introduced by inclusion of the *Xho*I restriction site; and purple, hexa-His tag. Nucleotide sequences were determined by Sanger sequencing (Eurofins) and are shown in the 5′ to 3′ direction.


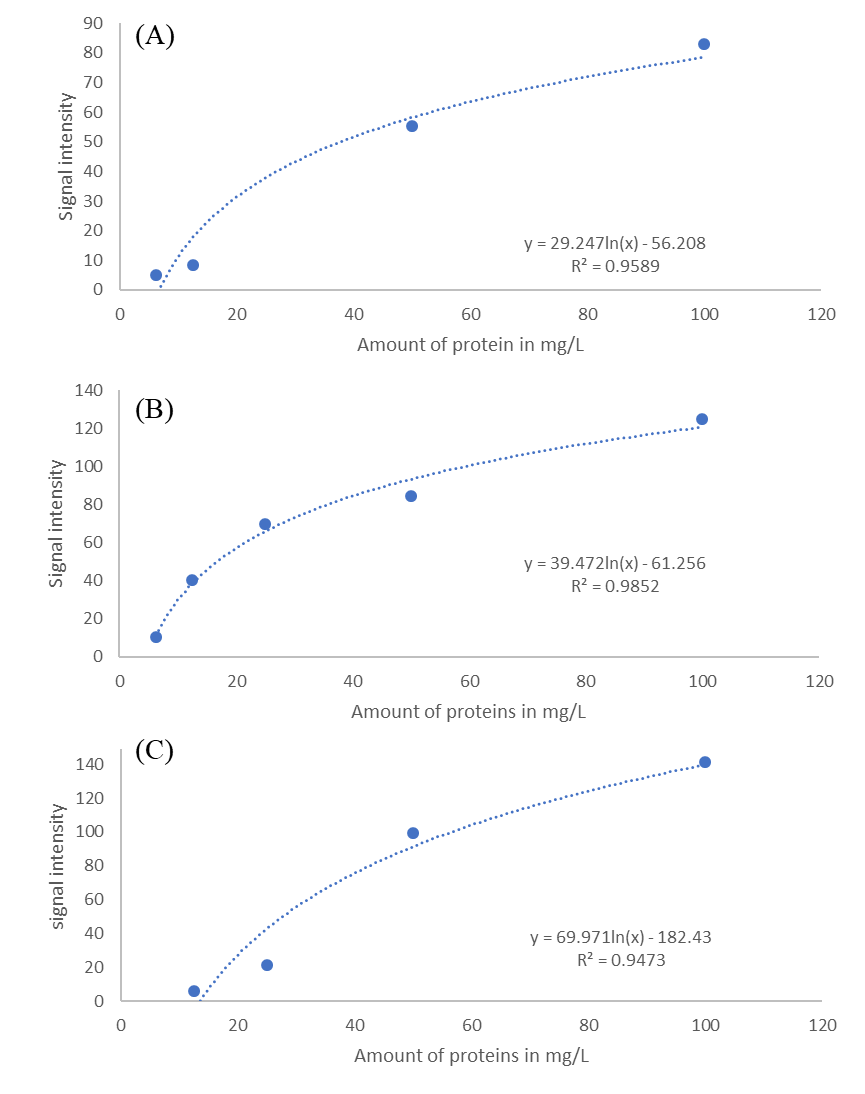


**Supplementary Figure 3.** Calibration curves plotted from signal intensity of bands obtained following western blot analysis (see Figure 1B). Calibration curves for Ex-FABP (A), Cal-γ (B) and α-1-ovoglycoprotein (C). The calibration was repeated for each quantification replication.


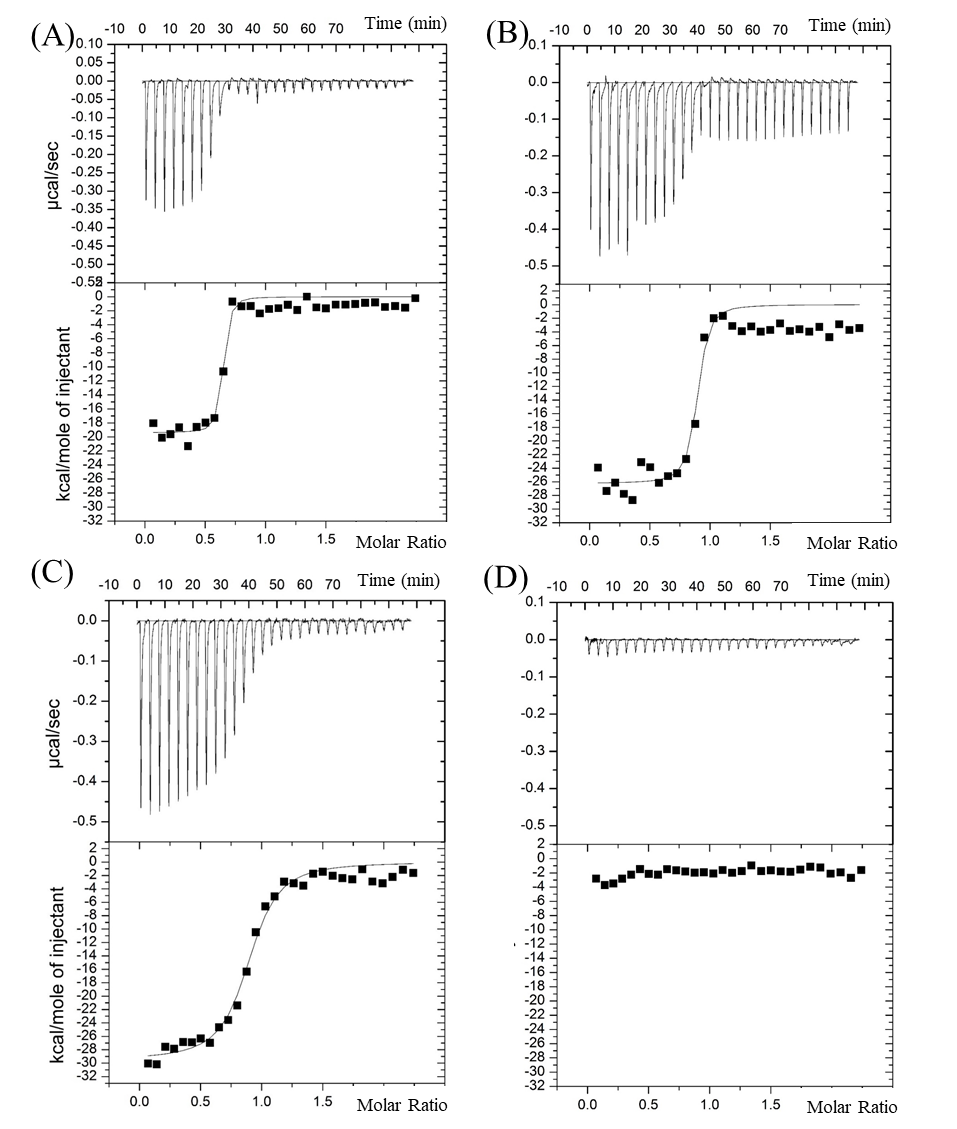


**Supplementary Figure 4.** Measurement of affinity of Ex-FABP for Fe-Ent (A), LCN2 for Fe-Ent (B), LCN2 for apo-Ent (C) and LCN2 for apo-Sal (DGE). Isothermal Titration Calorimetry was achieved with 29 injections (10 µL) of siderophore in an adiabatic well containing 5 µM of lipocalin. Experiments were achieved in TBS (20 mM Tris, 500 mM NaCl, 1.3% DMSO, pH 7.4) at 30°C.


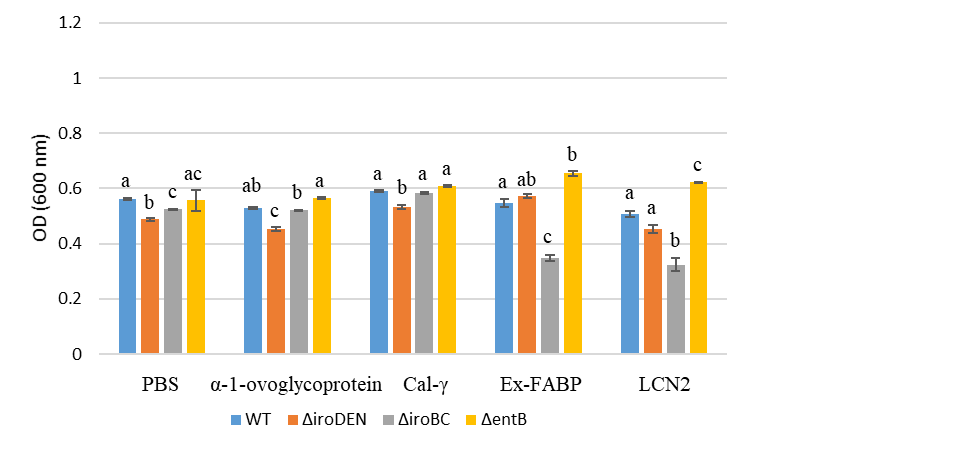


**Supplementary Figure 5.** Effect of EW lipocalins on growth of *Salmonella* Enteritidis and mutants defective in siderophore production and/or utilisation under iron restriction after 6 h growth. These data are derived from those presented in Fig. 4. Error bars indicate standard error from three biological replicates with two technical replicates**.** For each growth medium, one way-ANOVA followed by a multiple comparisons of means (Tukey Contrasts) was achieved using R software (version 3.5.3). This allowed to identify groups significantly different (identified as “a”, “b”, and “c”).

# Supplementary Tables

**Supplementary Table 1.** PCR primers used for deletion confirmation. Underscored amplicon sizes correspond to the expected size prior to gene knockout and bold amplicons sizes correspond to the expected size after gene knockout.

| Primer | Sequence 5’-3’ | Length (nucleotides) | Tm °C | Amplicons (bp) |
| --- | --- | --- | --- | --- |
| iroDEN_for | CGTAACCTGGCAAGGATGT | 19 | 63 | 4993 / **1159** |
| iroDEN_rev | GCCACGATTTACGCAAAA | 18 | 62.7 |  |
| iroBC_for | ATGATATTGGTAATTATTAT | 20 | 64.5 | 5157 / **1209** |
| iroBC_rev | CGTTAGTACTAATAGCTAAAGCGGC | 25 | 62.4 |  |
| entB_for | GCCGGTTGGTAAAGTCG | 17 | 62.2 | 1002 / **1177** |
| entB_rev | GCGTAACCGATCCCTTTC | 18 | 62.5 |  |
